# Study on site‐specific expression of bone formation and resorption factors in human dental follicles

**DOI:** 10.1111/eos.12568

**Published:** 2018-09-14

**Authors:** Pamela Uribe, Pawel Plakwicz, Lena Larsson, Ewa Czochrowska, Anna Westerlund, Maria Ransjö

**Affiliations:** ^1^ Department of Orthodontics Institute of Odontology Sahlgrenska Academy University of Gothenburg Gothenburg Sweden; ^2^ Department of Periodontology and Oral Mucosa Diseases Medical University of Warsaw Warsaw Poland; ^3^ Department of Periodontology Institute of Odontology Sahlgrenska Academy University of Gothenburg Gothenburg Sweden; ^4^ Department of Orthodontics Medical University of Warsaw Warsaw Poland

**Keywords:** connexin 43, osteoblast, osteoclast, receptor activator of nuclear factor‐kappa B ligand

## Abstract

We sought to investigate site‐specific expression of bone‐regulatory factors expressed by human dental follicles and to compare the stimulated expression of tumour necrosis factor (ligand) superfamily, member 11/tumour necrosis factor receptor superfamily, member 11b (*RANKL/OPG*) in human dental follicle cells (HDFCs) from different patients. Analysis of bone‐regulatory markers in follicles from 12 different study participants was performed using RT‐qPCR and immunofluorescence; apical and coronal segments from each dental follicle were processed independently. Four additional dental follicles were used for cell cultures; HDFCs were precultured in osteogenic medium to initiate differentiation and thereafter cultured with 10^−6^ M forskolin (FSK) to activate the protein kinase cAMP (PKA/cAMP) signalling pathway and induce *RANKL*/*OPG* expression. We demonstrate that *RANKL* expression is significantly higher in the coronal part of follicles than in the apical part. High levels of collagen type 1 (*COL1*)*,* alkaline phosphatase (*ALP*) and Gap‐junction protein, alpha 1, 43 kDa (*CX43*) were expressed, whereas expression of Sp7 transcription factor (*OSX)*, bone morphogenetic protein 2 (*BMP2*)*,* colony‐stimulating factor 1 (CSF‐1), chemokine (C‐C motif) ligand 2 (*MCP1*), and *OPG* was low in all samples. The immunofluorescence localization of *CSF‐1*,* MCP1*, osteocalcin (*OCN*)*, RANKL,* and *BMP2* was not specific for either part of the follicles. In conclusion, a consistently high expression of *CX43* suggests that gap‐junction communication in HDFCs is essential for the eruption process. Furthermore, the induced expression of *RANKL* in HDFCs varies significantly between individuals and may relate to clinical variations in tooth eruption.

Previous studies, looking at a variety of teeth and species, have suggested that the dental follicle plays a critical role in orchestrating the intraosseous component of tooth eruption 1, 2, 3. It has been postulated that the dental follicle displays regional differences in bone‐regulatory functions, that is, bone resorption and bone formation 4.

Expression of differentiation factors and signalling molecules essential for osteoclastogenesis and osteogenesis has been demonstrated in dental follicles obtained from rats and mice 1, 2, 3, 4, 5, 6, 7, 8, 9. Colony‐stimulating factor 1 (CSF‐1), chemokine (C‐C motif) ligand 2 (MCP‐1), tumour necrosis factor (ligand) superfamily, member 11 (RANKL), and tumour necrosis factor receptor superfamily, member 11b (OPG), which are key regulators of osteoclastogenesis, are differentially expressed in the coronal part of the dental follicle at specific stages during the eruption process 10, 11, 12. In addition, alveolar bone formation occurs in the basal region of the alveolar crypt. This may, in part, be stimulated by bone morphogenetic protein‐2 (BMP‐2) 13, 14.

The current body of experimental evidence from animal studies strongly suggests that the dental follicle is required for tooth eruption to occur normally in humans. However, the precise temporal and spatial patterns of gene expression that regulate site‐specific osteoclast and osteoblast activities during tooth eruption have not been studied in human follicles. Reaching a better understanding of the regulatory mechanisms and validating the putative component molecules are essential steps in elucidating eruption disorders. The aims of the present study were to investigate and compare the expression levels of regulatory factors in the human dental follicle to define local differences (i.e. within the follicles) regarding the expression of factors that regulate bone resorption and bone formation and to compare the stimulated expression of the osteoclast‐regulatory factors RANKL/OPG in human dental follicle cells (HDFCs) from different patients.

## Patient and methods

### Ethics

This study was approved by the Bioethics Committee at the Medical University of Warsaw (KB/124/2016) and by the Regional Ethics Board at the University of Gothenburg (Dnr. 898‐13). Informed consent was obtained from all the participating juvenile patients and their parents.

### Sampling

**Figure 1:**
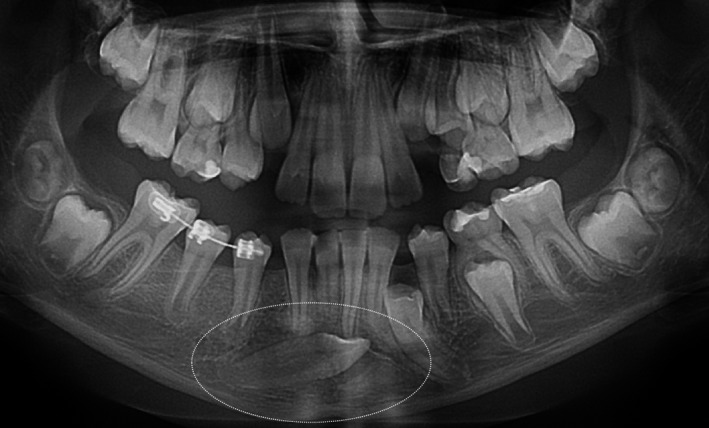
Radiograph showing an impacted canine tooth used for autotransplantation.

Patients with craniofacial syndromes, systemic metabolic diagnosis, or any bone disease were excluded from the study. Samples of dental follicles were collected during the surgical exposure of impacted teeth used for autotransplantation (Fig. 1). All surgical procedures were performed, by one of the authors (PP), in the Department of Periodontology at the Medical University of Warsaw and in a private dental practice in Warsaw. Nine female and three male patients (mean age, 13.3 yr; age range, 10–16 yr) were enrolled. In total, 12 dental follicles of permanent teeth were collected (Table 1). All the teeth were located intraosseously and entirely covered by bone, and all teeth had considerable root length. The collected specimens were free of gingival tissue.

**Table 1:** Description and distribution of the patients’ samples used for gene expression and immunofluorescence analyses

Patient no.	Age (yr)	Sex	Tooth	Apex	Sample no.	Area follicle
1	16	Male	13	Closed	1	Coronal
					2	Apical
2	16	Male	23	Closed	3	Coronal
					4	Apical
3	16	Female	23	Closed	5	Coronal
					6	Apical
4	11	Female	43	Open	7	Coronal
					8	Apical
5	12	Female	33	Closed	9	Coronal
					10	Apical
6	16	Female	23	Closed	11	Coronal
					12	Apical
7	10	Female	43	Open	13	Coronal
					14	Apical
8	13	Female	23	Closed	15	Coronal
					16	Apical
9	16	Female	23	Closed	17	Coronal
					18	Apical
10	10	Female	43	Open	19	Coronal
					20	Apical
11	12	Female	33	Open	21	Coronal
					22	Apical
12	12	Male	33	Open	23	Coronal
					24	Apical

In total, 24 samples of dental follicular tissues were collected from 12 patients. The samples correspond to the coronal and apical parts of the follicle, respectively, from each individual patient. Apex status was determined by clinical and radiographic examinations.

**Figure 2:**
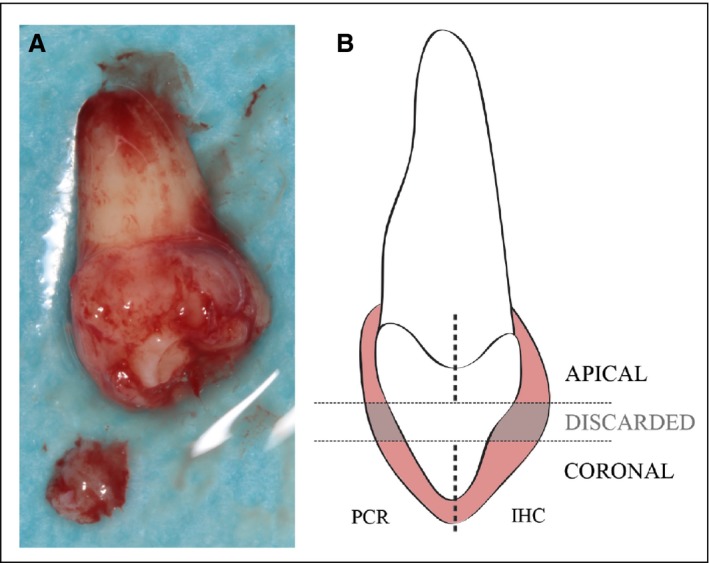
Illustration of the sampling process. (A) Dental follicle during the sampling procedure in which the coronal portion has been sectioned. (B) The dental follicle was dissected into three different fractions. The middle portion was discarded, and the coronal and apical portions were independently fixed. Subsequently, each of the apical and coronal parts was divided in half, with one half being used for gene‐expression analyses and the other half being used for immunofluorescence. IHC, immunohistochemistry.

Isolation of the coronal and apical portions of the dental follicle was performed after removal of the tooth from the bone crypt. The dental follicle was divided into three fractions (Fig. 2). The middle portion was discarded, and the coronal and apical portions were fixed separately in different containers. Thereafter, each piece was divided in half and placed in PAXgene tissue containers (PreAnalytiX, Hombrechtikon, Switzerland). PAXgene is a formalin‐free system designed to improve the quality of molecular analyses without diminishing the quality of histopathological analyses. The dual‐cavity containers are prefilled with fixation and stabilizer reagents. The specimens were fixed in PAXgene Tissue FIX Solution (PreAnalytiX) for 2–4 h and then transferred to the PAXgene tissue STABILIZER (PreAnalytiX) solution in the same container. Specimens were stored at −80°C until used in the gene‐expression and histology/immunofluorescence analyses.

### RT‐qPCR

The Minimum Information for Publication of Quantitative Real‐Time PCR Experiments (MIQE) guidelines were followed to ensure that the relevant experimental conditions and assay characteristics were followed 15. RNA extraction was carried out using the commercially available PAXgene Tissue RNA Kit (PreAnalytiX) for tissues and the RNeasy Plus Mini Kit (Qiagen, Hilden, Germany) for cells, according to the manufacturers’ instructions. Briefly, dental follicle tissue or cells were homogenized in the lysis buffer using a gentle MACS dissociator (Miltenyi Biotech, Bergish Gladbach, Germany). RNA was bound to the silica membrane using ethanol. The column was rinsed and washed with the TR2 and TR3 buffers provided, and then treated with DNase I to remove any contaminating DNA. The RNA was eluted and then stored at −20°C. The RNA concentrations were quantified using the Qubit Fluorometer (Invitrogen, Burlington, Ontario, Canada).

All reverse transcription steps were performed using the iScript cDNA Synthesis Kit (Bio‐Rad Laboratories, Hercules, CA, USA) with 1 *μ*g of total RNA. Two microliters of Universal RNA Spike (TATAA Biocenter, Gothenburg, Sweden) were added to each sample to allow quality control throughout the entire RT‐qPCR experimental workflow.

To select the most stable reference genes for normalization, a panel of 12 reference genes was screened in five samples of the retrieved tissues. The expression profiles of the screened reference genes were evaluated using geNorm 16 and Normfinder 17 computer programs. The reference genes peptidylprolyl isomerase A (*PPIA*) and glucuronidase, beta (*GUSB*) showed the most stable expression between the samples and were therefore selected as the reference genes in the subsequent analyses. The measured Cq value and the shape of the amplification curve reflected a lack of inhibition in the presence of RNA spiking in the control assays.

The primers used in the RT‐qPCR were purchased from Bio‐Rad Laboratories and are listed in Table 2. The target genes and the best two reference genes selected were analysed in a 10‐*μ*l reaction volume (10 ng of cDNA per reaction), in triplicate, in the CFX 96 Real‐Time System (Bio‐Rad Laboratories) using the SsoAdvanced Universal SYBR Green Supermix (Bio‐Rad Laboratories). An interplate calibrator (TATAA Biocenter) was added to each plate to compensate for the variation between runs. The quantities of the target genes were normalized using the geometric mean of the Cq values of the selected reference genes. Gene expression was quantified according to the comparative threshold cycle method delta‐delta Cq and 90% PCR efficiency 18. The results are presented as the mean value of three different experiments.

**Table 2:** Biorad SYBR Green primers used for the RT‐qPCR

Gene identification	Gene symbol	Unique assay ID	Amplicon context sequence 5′ to 3′
Target gene encoding			
Runt‐related transcription factor 2	*RUNX2*	qHsaCED0044067	TTCCTCATCCCAGTATGAGAGTAGGTGTCCCGCCTCAGAACCCACGGCCCTCCCTGAACTCTGCACCAA GTCCTTTTAATCCACAAGGACAGAGTCAGATTACAGACCCCAGG
Sp7 transcription factor	*OSX*	qHsaCED0003759	AGTTTGTGGAAAGCCAGTCTCATGGTGAAGAGAGAAGGTGAGCTGAGGAGGCATGCAAGGAGATAC CCAAGCCCAGAATGGCCAGGAGGGAGACAGAGGGAGAGAGCCCCGAAGGATGGCATGCATGGGGT AAAGAGAGTGATTGGCAAGCAGTGGTCTAGAGAGCCAA
Bone morphogenetic protein 2	*BMP2*	qHsaCID0015400	GCCAGGTCCTTTGACCAGAGTTTTTCCATGTGGACGCTCTTTCAATGGACGTGTCCCCGCGTGCTTCTT AGACGGACTGCGGTCTCCTAAAGGTCGACCATGGTGGCCGGGACCCGCTGTCTTCTAGCGTTGCTGCTTCCCC
Alkaline phosphatase	*ALP*	qHsaCID0010031	CGCCTACCAGCTCATGCATAACATCAGGGACATTGACGTGATCATGGGGGGTGGCCGGAAATACA TGTACCCCAAGAATAAAACTGATGTGGAGTATGAGAGTGACGAGAAAGCCAGGGGCACGAGGCT
Collagen type I	*COL1*	qHsaCED0002181	CCCCCGCATGGGTCTTCAAGCAAGTGGACCAAGCTTCCTTTTTTAAAAAGTTATTTATTTATTCTTTT TTTTTTTTTTTTTTTGGTAAGGTTGAATGCACTTTTGGTTTTTGGTCATGTTCGGTTGGTCAAAGATAAAAACTAA
Gap‐junction protein, alpha 1, 43 kDa	*CX43*	qHsaCID0012977	CTTCATCCTCCAAGGAGTTCAATCACTTGGCGTGACTTCACTACTTTTAAGCAAA AGAGTGGTGCCC AGGCAACATGGGTGACTGGAGCGCCTTAGGCAAACTCCTTG ACAAGGTTCAAGCCTACTCAACTGC TGGAGGGAAGGTGTGGCTGTCAGTACT
Tumour necrosis factor receptor superfamily, memb.11b	*OPG*	qHsaCED0046251	TACTCATCCATGGGATCTCGCCAATTGTGAGGAAACAGCTCAATGGCCATTTCCAGTTATAAGCAGCTT ATTTTTACTGATTGGACCTGGTTACCTATCATTTCTAAAAATAAC
Tumour necrosis factor (ligand) superfamily, member 11	*RANKL*	qHsaCID0015585	CGATGGTGGATGGCTCATGGTTAGATCTGGCCAAGAGGAGCAAGCTTGAAGCTCAGCCTTTTGCTCATCTCACTA TTAATGCCACCGACATCCCATCTGGTTCCCATAAAGTGAGTCTGTCCTCTTGGTACCATGATCGGGGTTGGGC
Chemokine (C‐C motif) ligand 2 (CCL2)	*MCP1*	qHsaCID0011608	ACTGAAGCTCGCACTCTCGCCTCCAGCATGAAAGTCTCTGCCGCCCTTCTGTGC CTGCTGCTCATAGCAGCCACCT TCATTCCCCAAGGGCTCGCTCAGCCAGATGCA ATCAATGCCCCAGTCACCTGCTGTTATAACTTCACCAATAGGAA GATCTCAGTGC AGAGGCTCGCGAGCTAT
Colony‐stimulating factor 1 (macrophage)	*CSF1*	qHsaCID0016847	GGAGACCTCGTGCCAAATTACATTTGAGTTTGTAGACCAGGAACAGTTGAAAGATCCAGTGTGCTACCTTAAGAAGG CATTTCTCCTGGTACAAGA
Reference gene encoding:			
Glucuronidase, beta	*GUSB*	qHsaCID0011706	ACCAAGAGTAGTAGCTGTTCAAACAGATCACATCCACATACGGAGCCCCCTTGT CTGCTGCATAGTTAGAGTTGCTCACAAAGGT CACAGGCCGGGAGGGGTCCAAG GA
Peptidylprolyl isomerase A (cyclophilin A)	*PPIA*	qHsaCED0038620	CTCGAATAAGTTTGACTTGTGTTTTATCTTAACCACCAGATCATTCCTTCTGTAGCTCAGGAGAGCACCCCTCCACCC CATTTGCTCGCAGTATCCTAGAATCTTTGTGCTCTCGCTGCAGTTCCCTTTGGGTTCCATGTTTTCCTTGTTCCC TCCCATGCCT

### Immunohistochemistry/Immunofluorescence

Twelve specimens were fixed in the PAXgene solutions. One‐half of each specimen was sent to Biobanken Norr (Umeå, Sweden) for standard embedding and sectioning. Fixed tissues were placed in embedding cassettes and subjected to progressive dehydration in an ethanol dilution series. Subsequently, the tissues were exposed to xylene three times before being embedded in paraffin blocks. Serial sections of 4 *μ*m thickness were cut at three different levels, and the sections were split onto three separate slides.

The PAXgene‐fixed and paraffin‐embedded tissue samples were washed in xylene and a dilution series of ethanol, then treated with 1 × Diva Decloaker heat retrieval solution (Biocare Medical, Concord, CA, USA), at pH 6.5 and 65°C overnight, to reveal the epitopes. After washing with PBS, the sections were blocked with 3% BSA for 45 min before incubation overnight at 4°C with the following primary antibodies (Abcam, Cambridge, UK): anti‐(CSF‐1) (ab9693; diluted 1:400); anti‐osteocalcin (anti‐OCN; ab13420; diluted 1:350) anti‐(Gap‐junction protein, alpha 1, 43 kDa) (anti‐CX43; ab87645; diluted 1:350); anti‐(BMP‐2) (ab14933; diluted 1:350); and anti‐RANKL (ab45039; diluted 1:300). After washing with PBS, the sections were incubated with the secondary antibodies (Invitrogen, Thermo Fisher Scientific) for CSF‐1 (A11011; anti‐rabbit Alexa 568‐conjugated; diluted 1:200), OCN (A21050; anti‐mouse Alexa 633‐conjugated; diluted 1:50), CX43 (A21222; anti‐goat Alexa 488‐conjugated; diluted 1:200), BMP2 (A11011; anti‐rabbit Alexa 568‐conjugated; diluted 1:200), and RANKL (A21050; anti‐mouse Alexa 633‐conjugated; diluted 1:50) for 2 h at room temperature, followed by 3 min of treatment with HOECHST solution (H3570) (Invitrogen, Thermo Fisher Scientific) for visualization of cell nuclei, before mounting with Fluoroshield mounting medium (Sigma‐Aldrich, St Louis, MO, USA). Several control steps were performed to ascertain the specificity of the antibodies, including incubation with only the blocking agent, only the primary antibody, only the secondary antibody, and only the tissue. The fluorescence images were captured using an LSM 710 NLO microscope from Carl Zeiss (Oberkochen, Germany).

### Cell culture

Primary cultures of human dental follicle cells (HDFCs) were established using the dental follicles obtained from four different patients presenting impacted canines and referred for surgical exposure.

After rinsing in MEM Alpha medium 1 × (*α*‐MEM; Gibco Life Technologies, Grand Island, NY, USA), tissues were minced using a sterile scalpel, cultured in *α*‐MEM supplemented with 10% (v/v) fetal bovine serum (Gibco Life Technologies), 2 mM Glutamax (Gibco Life Technologies), and Antibiotic‐Antimycotic reagent (Gibco Life Technologies) at 37°C in humidified air with 5% CO_2_. After 48 h, non‐adherent cells were removed by changing the medium. Human dental follicle cells used at passages 3–5 were cultured in *α*‐MEM with or without osteogenic induction medium (50 mg ml^−1^ of L‐ascorbic acid 2‐phosphate sesqui‐magnesium salt and 10 mM *β*‐glycerophosphate disodium salt hydrate) (Sigma‐Aldrich). After 14 d, HDFCs were cultured in *α*‐MEM containing 10^−6^ M forskolin (FSK; Sigma‐Aldrich) for 0, 24, and 48 h; the cells at the 0‐h time point were defined as controls. The FSK concentration was obtained from dose–response studies previously performed 19, 20.

The results are presented as the average of two different experiments performed in duplicate. The relative levels of *RANKL* and *OPG* transcription in FSK‐treated HDFCs were adjusted by standardization based on the levels of *GUSB* mRNA.

### Statistical analysis

Gene‐expression data were analysed using the PRISM 7 software package (GraphPad Software, San Diego, CA, USA). The coronal and apical sections of the follicles were analysed using the Wilcoxon signed‐rank test to determine significant differences in gene expression between matched pairs of samples; values of *P* ≤ 0.05 were considered to be statistically significant.

## Results

### Gene‐expression analysis of coronal and apical follicle samples

**Figure 3:**
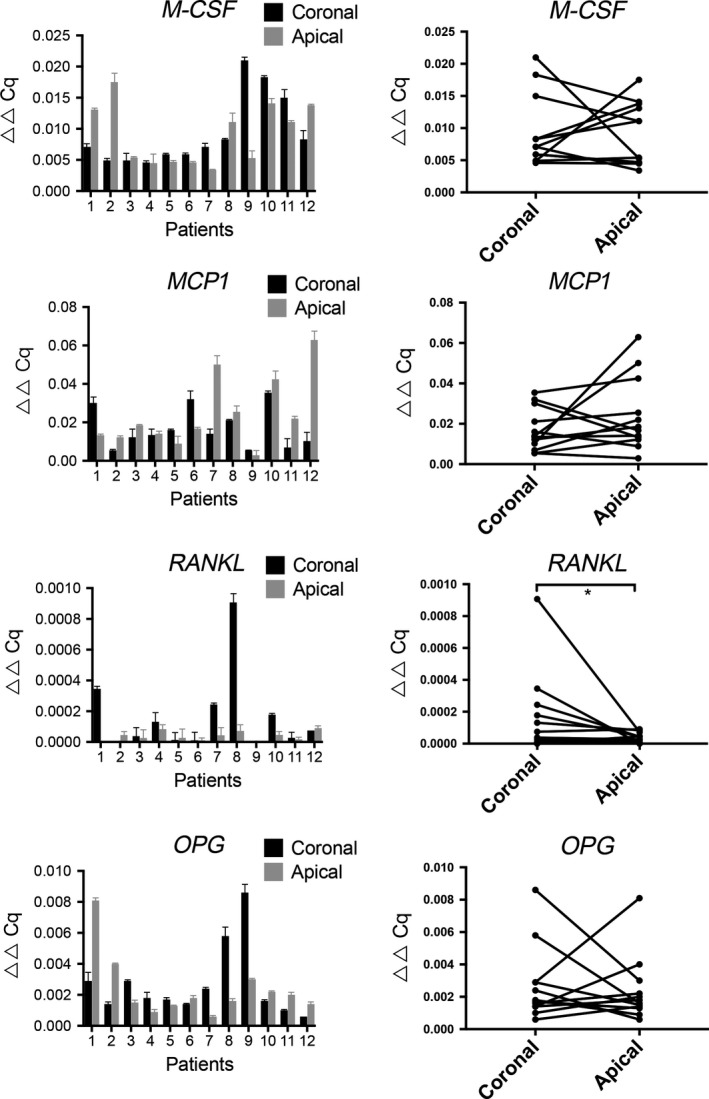
Comparison of gene‐expression profiles in the coronal and apical portions of the dental follicle. The relative expression levels of the indicated target genes related to markers of bone resorption in the dental follicle are shown. The data are presented as ▵▵Cq values in relation to the relative gene‐expression level of the selected reference gene [peptidylprolyl isomerase A (*PPIA*)] ± SEM. The values shown in the charts in the right‐hand column correspond to the values obtained for coronal and apical sections of the follicles following analysis using the Wilcoxon signed‐rank test to determine significant differences in gene expression between matched pairs of samples; values of *P* < 0.05 were considered to be statistically significant. *MCP1*, chemokine (C‐C motif) ligand 2; *CSF‐1*, colony‐stimulating factor 1; *OPG*, tumour necrosis factor receptor superfamily, member 11b; *RANKL*, tumour necrosis factor (ligand) superfamily, member 11.

The relative transcription levels of the osteoclast‐related markers *CSF‐1*,* MCP1*,* OPG*, and *RANKL* were low in all the samples. Notably, a significantly higher expression of RANKL was detected in the coronal parts of the dental follicles compared with the corresponding apical parts (*P* = 0.031). However, no specific expression patterns of *CSF‐1*,* MCP1*, or *OPG* were related to the apical or coronal parts of the dental follicles (Fig. 3).

**Figure 4:**
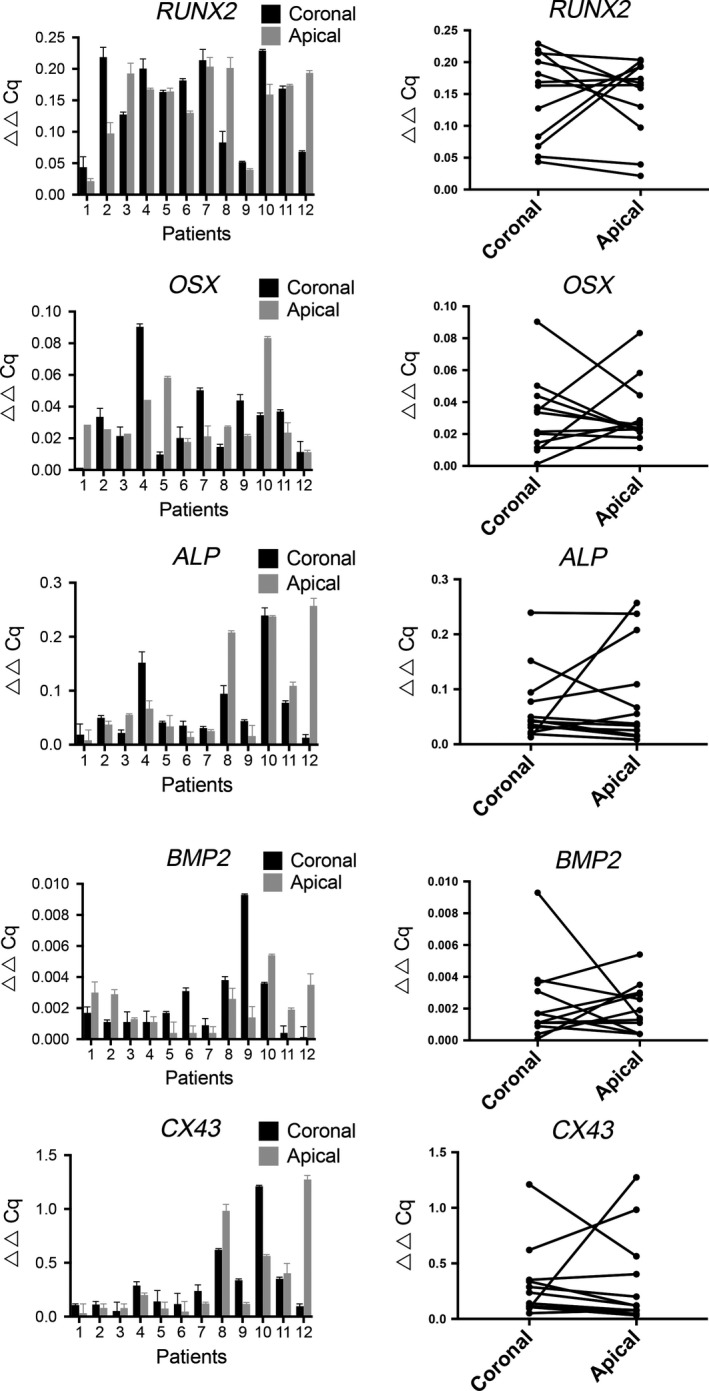
Comparison of gene‐expression profiles of the coronal and apical portions of the dental follicle. The relative expression levels of the indicated target genes related to markers of bone formation in the dental follicle are shown. The data are presented as ▵▵Cq values in relation to the relative gene expression level of the selected reference gene [peptidylprolyl isomerase A (*PPIA*)] ± SEM. The values shown in the charts in the right‐hand column correspond to the values obtained for coronal and apical sections of the follicles following analysis using the Wilcoxon signed‐rank test to determine significant differences in gene expression between matched pairs of samples; values of *P* < 0.05 were considered to be statistically significant. *ALP*, alkaline phosphatase; *BMP2*, bone morphogenetic protein 2; *CX43*, gap‐junction protein, alpha 1, 43 kDa; *OSX*, Sp7 transcription factor; *RUNX2*, runt‐related transcription factor 2.

Examining the expression levels of runt‐related transcription factor 2 (*RUNX2*), Sp7 transcription factor (*OSX*), *CX43*,* BMP2,* and alkaline phosphatase (*ALP*), which are osteoblast‐associated transcription factors and differentiation markers, allowed us to assess the regulation of new bone formation and to determine whether the expression of these factors was higher in the apical part of the dental follicles (Fig. 4). The levels of *OSX* and *BMP2* were relatively low in all samples. The highest levels of expression were found for collagen type 1 (*COL1*) and *ALP*. Furthermore, *CX43* (the gene that encodes gap‐junction protein) was strongly expressed in all the specimens evaluated. Differences in expression levels were detected both in the samples from different patients and when comparing the expression profiles of the target genes. Interestingly, a pattern was revealed in which the same samples displayed expressed high levels of *COL1*,* BMP‐2*,* ALP*, and *CX43*. However, there was no specific linkage to higher expression of these particular genes in the apical parts of the dental follicles compared with expression in the corresponding coronal parts.

In addition, we included three dental follicles from teeth that erupted normally in parallel with the analysed samples (data not shown). When comparing the normally erupting teeth with the dental follicles from impacted teeth, no statistically significant differences were observed in the expression levels of the selected bone markers.

### Immunofluorescence staining of follicular tissues

**Figure 5:**
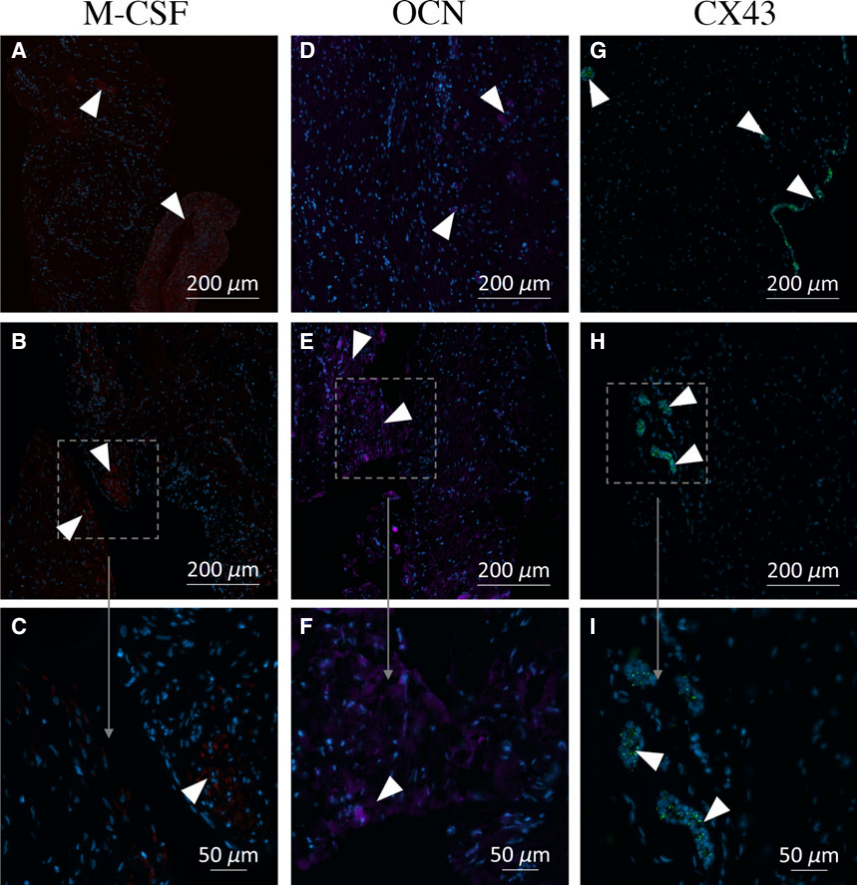
Immunofluorescence photomicrographs of osteocalcin (OCN), gap‐junction protein, alpha 1, 43 kDa (CX43), and colony‐stimulating factor 1 (CSF‐1) expression in sections of dental follicle. The localizations of CSF‐1 (red), OCN (purple), and CX43 (green) are shown in representative images of sections of the human dental follicle obtained from different patients. No obvious spatial pattern of expression of the proteins is discernible. Staining with antibodies directed against bone morphogenetic protein 2 (BMP‐2) and RANKL was negative for all the samples. Positive areas are enlarged for a more detailed evaluation. The sections examined are from the following patients and portions of the follicle: (A) Patient 1, coronal; (B, C) Patient 3, apical; (D) Patient 3, apical; (E, F) Patient 1, coronal; (G) Patient 5, apical; (H, I) Patient 2, coronal.

To elucidate the spatial expression patterns of CSF‐1, OCN, CX43, BMP‐2, and RANKL in the dental follicle sections, immunofluorescence staining was performed. Confocal microscopy images of the tissue sections revealed specific expression of CSF‐1, OCN, and CX43. No specific staining for BMP‐2 or RANKL was observed in any of the tissues. Visual comparison of the stained specimens showed no specific localization pattern for the bone‐resorption or bone‐formation markers for either the apical or the coronal segments; representative images of positive and specific staining are presented in Fig. 5.

### Gene‐expression analysis in activated HDFCs

**Figure 6:**
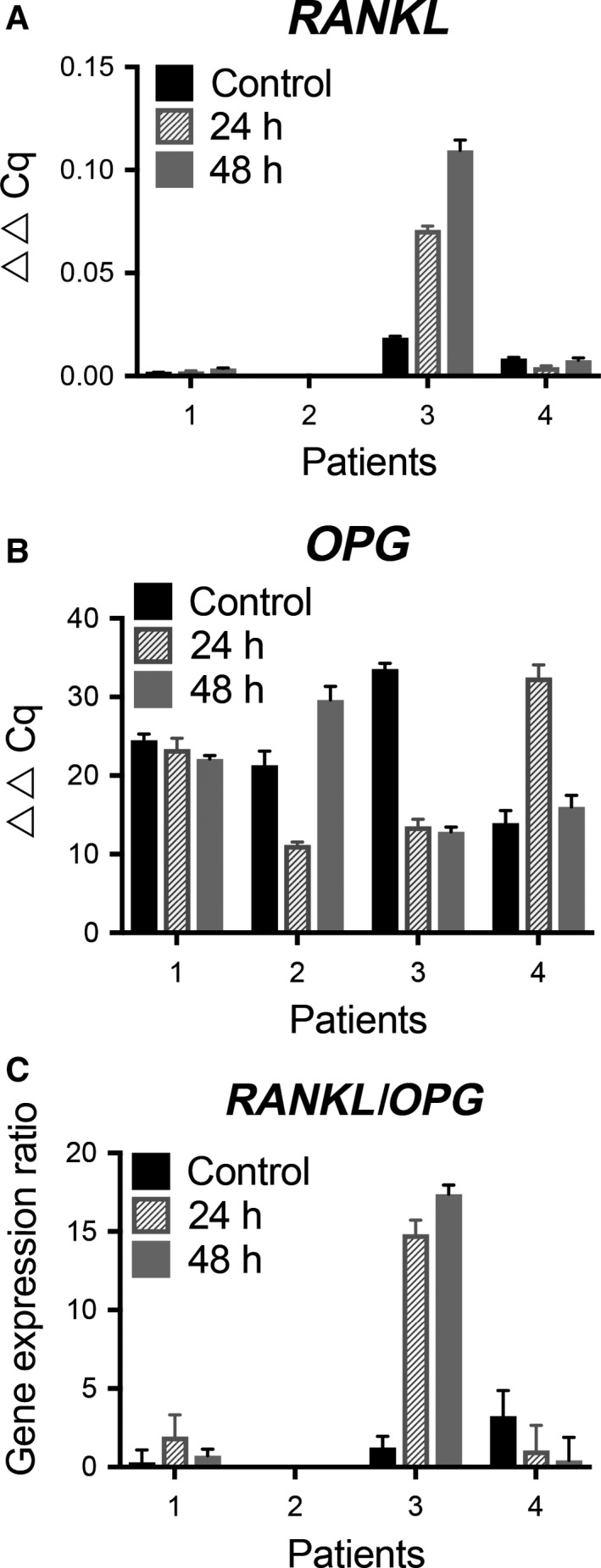
Effects of forskolin (FSK) on tumour necrosis factor (ligand) superfamily, member 11 (*RANKL*) and tumour necrosis factor receptor superfamily, member 11b (*OPG*) gene expression levels in cultured human dental follicle cells (HDFCs). The HDFCs from four patients were precultured in MEM Alpha medium (*α*‐MEM) with or without the presence of osteogenic induction medium for 14 d and thereafter cultured in *α*‐MEM with FSK (10^−6^ M) for the stated time periods. The results are presented as the average of two different experiments performed in duplicate. The expression levels of *RANKL* (A) and *OPG* (B), measured by RT‐qPCR, are adjusted by standardization based on the expression levels of glucuronidase, beta (*GUSB*) and are presented as fold increases relative to the control over time (0, 24, and 48 h). The *RANKL/OPG* ratios are presented as mean ± SEM (*n* = 5). (C) Ratios of *RANKL/OPG* gene expression are presented as ▵▵Ct values.

We used the adenylate cyclase activator FSK to explore whether HDFCs obtained from various patients exhibit a differential capacity for regulating expression of the *RANKL* and *OPG* genes. The HDFCs from four different patients were first cultured in osteogenic medium to initiate differentiation and were thereafter cultured in the presence of 10^−6^ M FSK. Notably, FSK treatment induced substantial variation in the level of stimulated *RANKL* expression in the follicle cells from different patients, suggesting a completely different regulatory pattern (Fig. 6).

The *OPG* gene was highly expressed in the cultured control cells derived from all patients before activation with FSK. No clear time‐dependent regulatory effect on *OPG* expression was seen after 24 and 48 h in culture. There were significant variations in the *RANKL/OPG* expression ratios but no differences in the regulatory patterns between patients (Fig. 6).

## Discussion

This study is the first to evaluate site‐specific expression of regulatory factors in the human dental follicle. Our results demonstrate expression/upregulation of important osteoblast‐ and osteoclast‐associated markers. However, significant differential spatial gene expression could only be demonstrated for *RANKL* in the coronal part of the dental follicle, compared with the corresponding apical part. This is in agreement with the results of earlier studies in dogs, as presented by marks & cahill 6 and wise *et al*. 14. In contrast, the relative transcription levels of *CSF‐1*,* MCP‐1*, and *OPG* were low in all the dental follicle samples, with no specific pattern being related to the apical or coronal parts of the dental follicle.

RANKL plays a critical role in tooth eruption, as demonstrated by the failure of teeth to erupt in *RANKL* gene‐knockout mice and osteopetrotic rodents 21. RANKL is expressed in response to various bone‐resorbing factors, for example, parathyroid hormone‐related protein (PTHrP). RANKL binds to its receptor RANK, which is expressed on both osteoclast progenitor cells and osteoclasts. The RANKL/RANK interaction is blocked by OPG, which is a decoy for RANKL and prevents its binding to the RANK receptor. The ratio of RANKL to OPG defines the extent of RANKL‐stimulated osteoclast recruitment and bone resorption 22. Thus, our demonstration of *RANKL* expression in the coronal portion of the dental follicle provides further support for the idea of RANKL as an important regulator of the osteoclastogenesis and bone resorption needed for formation of the tooth eruption pathway.

Previous studies in animals have suggested that bone formation at the base of the tooth crypt is required for the tooth to erupt 4, 6, 9, 14. The present study reveals that the human dental follicle expresses *RUNX2, OSX, BMP2,* and* ALP.* However, no specific pattern of osteoblast/bone‐formation markers associated with the apical portions of the dental follicle could be demonstrated. A possible explanation for the divergent results between our study and the aforementioned studies is that the apical part of the dental follicle is not relevant in later stages in tooth eruption because the dental follicle is not active after tooth‐root formation. The transcription factor RUNX2 can direct cells with differentiation potential to an osteoblastic lineage and prompt osteogenesis 23. The OSX protein has been isolated in mice and shown to be a specific regulator of osteoblast differentiation acting downstream of RUNX2 24. Notably, patients with cleidocranial dysplasia (CCD) have impaired tooth eruption and *RUNX2* mutations. It has been reported that dental follicle from patients with CCD has altered ratios of *RANK/RANKL* and *RANKL/OPG* expression, which result in decreased osteoclastogenesis and bone resorption, thus ultimately impairing tooth eruption 25, 26. The present study reveals that human dental follicles express *RUNX2, OSX, BMP2*, and *ALP*.

Unlike our clinical samples from patients, the sampling in the animal experimental studies could be time‐controlled to take place earlier during the eruption process. Most of the molecular data on tooth eruption collected to date have been obtained from studies conducted in rodents and dogs. The use of animals to model humans in biological development relies on the notion that basic processes are sufficiently similar to allow extrapolation. However, projection of these data to humans should be approached with caution, given the known differences between these diverse species. As a result of ethical considerations, human dental follicles can only be obtained in specific circumstances, such as in cases of impacted canines and third molars. The lack of a control group is the major limitation of the present study.

The results of the present study demonstrate strong and continuous regulation of the gap‐junction protein CX43 in all the dental follicle samples. Gap‐junction communication, which plays an important role in the growth and differentiation of many tissues and cells, is a cell‐to‐cell signalling system that permits the transfer of small molecules, ions, and second messengers between adjacent cells. Gap‐junction channels in bone, mainly composed of CX43 subunits, are crucially important for osteoblast differentiation, the formation of the bone matrix, and mineralization 27, 28. In addition, osteoclast precursors and mature osteoclasts are reported to express the CX43 protein and functional gap‐junction channels necessary for their development and bone‐resorbing activities 27, 29, 30. The high‐level expression of *CX43* in dental follicles has not been reported before and suggests that signals that are dependent upon gap‐junction communication may be essential for the regulatory functions active during the eruption process.

It has previously been reported that RANKL can be induced in cultured HDFCs by extracellular factors, for example, PTHrP 31. The cAMP/protein kinase A (PKA) pathway is one of the major signalling pathways for the expression of *RANKL* 20. The FSK protein is a potent adenylyl cyclase activator which increases the level of second messenger cAMP in bone cells 19. Given this, we used a cell culture approach and FSK as an experimental tool to investigate whether stimulated expression of *RANKL* in HDFCs differed among the patients. The in vitro‐induced expression of *RANKL* demonstrates that there are significant differences between individuals in relation to the levels of *RANKL* expression, the responses to external stimuli, and the control of the *OPG/RANKL* ratio (with respect to favouring or limiting osteoclastic activity). Although based on a limited number of follicle samples, our findings may have relevance for the diversity of events that are noted in the clinical setting during tooth eruption.

The results of this study demonstrate a significant local differential expression of the osteoclast regulator RANKL in human dental follicles. Moreover, a contrasting stimulated RANKL response is shown in HDFCs from different patients. This may, in turn, explain the differences during eruption events observed in the clinic. The consistent expression of *CX43* indicates that signals related to gap‐junction communication are essential for the eruption process.

Insight into the eruption process is important, not only for determining the mechanisms underlying normal eruption of teeth in humans, but may also lead to reference data when studying eruption disturbances in humans in future studies.

## Conflicts of interest

All of the authors declare no potential conflict of interests with respect to the authorship and/or publication of this article.
